# Comparative global burden analysis of lymphoma subtypes: a statistical evaluation of severity across global regions

**DOI:** 10.3389/fpubh.2025.1590093

**Published:** 2025-04-25

**Authors:** Yu Liang, Yingying Tang, Yuanyuan WanYan, Erwei Li

**Affiliations:** ^1^Department of Blood Transfusion, Henan Provincial People’s Hospital, Zhengzhou, Henan, China; ^2^Department of Blood Transfusion of Fuwai Central China Cardiovascular Hospital, Central China Fuwai Hospital of Zhengzhou University, Zhengzhou, Henan, China

**Keywords:** lymphoma, Hodgkin lymphoma, non-Hodgkin lymphoma, Burkitt lymphoma, global health burden

## Abstract

**Introduction:**

Various subtypes of lymphoma, including Hodgkin lymphoma, non-Hodgkin lymphoma, and Burkitt lymphoma, impose a significant global health burden. This study aimed to analyze the severity of these lymphoma types across different global regions from 1990 to 2021, focusing on age-standardized incidence rates, disability-adjusted life years (DALYs), and the Quality of Care Index (QCI).

**Methods:**

Data were collected from the Institute for Health Metrics and Evaluation (IHME). Global regions were categorized according to their socio-demographic index (SDI), and trends in death rates, DALYs, and case numbers of various lymphoma subtypes were examined over a 31-year period. The Analysis of Variance (ANOVA) test was employed to compare mortality and DALYs among regions.

**Results:**

The analysis revealed significant global and regional trends in lymphoma burden. For Hodgkin lymphoma, DALYs decreased from 0.000513 to 0.000285, and the death rate declined from 0.000629 to 0.000313. In contrast, non-Hodgkin lymphoma exhibited an increase in DALYs from 0.002012 to 0.002430 and in deaths from 0.003182 to 0.003624. Notably, in low SDI regions, a marked increase in DALYs was observed for Hodgkin lymphoma (from 0.000396 to 0.000922) and non-Hodgkin lymphoma (from 0.001147 to 0.002779), with statistical analyses confirming higher mortality rates in these areas.

**Discussion:**

These findings highlight significant regional disparities in the severity of lymphoma. While high-income regions demonstrated lower mortality rates due to better healthcare infrastructure, low-income areas experienced higher mortality, underlining the inequality in healthcare access. The results underscore the urgent need for targeted healthcare interventions and specialized treatment strategies to reduce the global lymphoma burden, particularly in under-resourced regions.

## 1 Introduction

Lymphomas, including Hodgkin lymphoma, non-Hodgkin lymphoma and Burkitt lymphoma are serious diseases of the lymphatic system and are recognized as a global cancer concern. The incidence of these types of lymphomas is on the rise significantly impacting environmental, social and economic conditions worldwide (1). Hodgkin lymphoma is characterized by the abnormal and malignant division of lymphocytes specifically affecting Reed-Sternberg cells. Lymphoma is most common across the globe and appears as symptoms such as pain, swelling, and weight loss at sudden (2). Early detection can lead to successful control, managing Hodgkin lymphoma remains a challenging task in especially in those regions having low grade medical facilities (3). Non-Hodgkin lymphoma (NHL) is a form of blood cancer with a rapidly increasing incidence and mortality rate. The highest number of NHL cases has been reported in Colombia, Eastern Europe, and Central Asia (4). Radiotherapy is a common treatment; however, the disease is often diagnosed in its early stages. As the disease progresses, it leads to swelling and pain in various parts of the body. Difficulties of NHL are exacerbated by conditions such as AIDS and immune-suppressive therapies (5). Burkitt lymphoma is an extremely aggressive subtype of non-Hodgkin lymphoma. Those affected are more susceptible to diseases like Epstein–Barr virus and malaria. Burkitt lymphoma has a high mortality rate in those regions having average limited healthcare system (6). The highest prevalence of Burkitt lymphoma is currently observed in sub-Saharan Africa. Despite extensive study on the subtypes of lymphoma, previous studies have typically focused on single locations or specific subtypes, limiting the understanding of the full scope and impact of lymphoma (7). Presented study addresses this gap through a comparative analysis of Hodgkin lymphoma, non-Hodgkin lymphoma, and Burkitt lymphoma globally and also at socio-demographic levels. Statistical approaches and long-term pattern analyses were employed to explore the causes of geographic variation in lymphoma outcomes for comprehensive understanding of lymphoma’s global impact (8). The findings from this study are poised to guide health policymakers in identifying critical areas where resources need to be allocated more effectively. This could significantly influence global health policies and research efforts aimed at reducing disparities in lymphoma severity between high and low-income regions (9). Furthermore, the study underscores the importance of future research into the socioeconomic and environmental factors influencing lymphoma outcomes, which could contribute to the development of evidence-based policies for improving global health outcomes related to lymphoma (10).

## 2 Method

**Figure 1:**
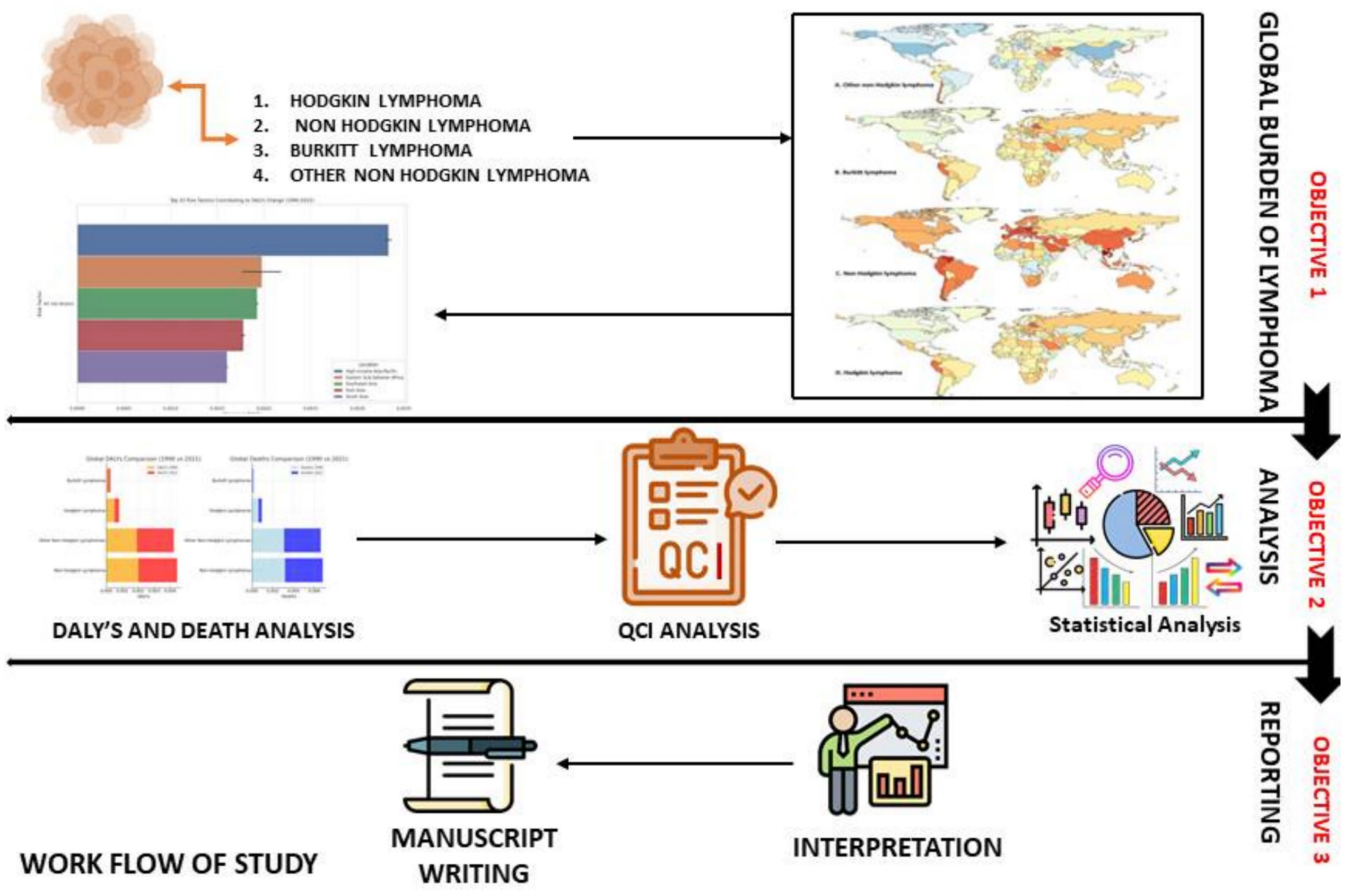
Workflow applied for this study. Objective 1–3 shows the three phases of work flow for this study.

The entire methodological workflow for the study is outlined in Figure 1. The study design, objectives, data collection, analysis, and reporting are detailed in the subsequent sections of the methodology.

### 2.1 Study design and objectives

Major emphasis for this study was set to evaluate the global burden of lymphoma by analysing their different subtypes, including Hodgkin lymphoma, non-Hodgkin lymphoma, Burkitt lymphoma and other non-Hodgkin lymphomas, over the period from 1990 to 2021 (11). During the study we identified trends in incidence, disability-adjusted life years (DALYs), mortality and Quality of Care Index (QCI) across different global regions classified by socio-demographic index (SDI) levels (12). A secondary objective was to explore the underlying factors contributing to these trends by analysing regional variations.

### 2.2 Data sources and study population

We obtained epidemiological information data for all four subtypes of lymphomas from IHME (Institute for Health Metrics and Evaluation: https://www.healthdata.org/research-analysis/gbd) (13). The study included age-standardized incidence rates, DALYs, mortality rates, and QCI as primary variables. Regions were classified based on their SDI (a combined measure reflecting per capita income, educational achievement and fertility rates) to group countries into low, low-medium, medium, medium-high and high-SDI categories. The study population encompassed all recorded incidences of lymphoma types, both sexes and all age groups between 1990 and 2021. Missing, incomplete, inconsistent, unreliable data from this study were excluded from the current analysis.

### 2.3 Variables and outcome measures

The primary variables in this study included age-standardized incidence rates of lymphoma, DALYs, mortality rates, and QCI. We standardized the incidence rate for age to facilitate comparisons across regions with varying age distributions. DALYs combined the years lost due to early mortality and years lived with a disability resulting from lymphomas. For analysis, mortality rate was measured per 100,000 individuals and QCI was used to evaluate the quality of healthcare services in different regions, with higher scores indicating better care. Secondary variables included the SDI and regional classifications to assess inequalities across geographic regions such as sub-Saharan Africa, Central Asia and high-income North America.

Screening of variables: to ensure the robustness and validity of our analysis, a systematic screening process was implemented for all variables extracted from the IHME database. Initially, a comprehensive list of potential variables related to the global burden of lymphoma—including age-standardized incidence rates, DALYs, mortality rates, and the QCI—was compiled. The selection of these variables was guided by their established relevance in previous epidemiological studies and their direct association with lymphoma outcomes. Subsequently, each variable was evaluated based on three main criteria: data completeness over the study period (1990–2021), consistency across different global regions, and comparability for meaningful statistical analysis. Variables exhibiting significant missing data or inconsistent reporting were excluded from the final analysis. In addition, secondary variables such as the SDI were screened to ensure they adequately stratified regions by socioeconomic status. This rigorous screening ensured that only reliable and pertinent data were incorporated into the subsequent statistical evaluations.

### 2.4 Data analysis

We calculated descriptive statistics to investigate the distribution of lymphoma incidence, DALYs, mortality, and QCI across various regions and SDI levels. We calculated the mean, median, standard deviation, and interquartile ranges for each variable. Trend analysis involved calculating annual percentage changes to identify regions with significant increases or decreases in lymphoma burden with time-series methods used to analyse trends in DALYs and mortality over the 31-year period. Visualization techniques such as line graphs, bar charts and heatmaps were employed to represent trends visually. Inferential analysis included Analysis of Variance (ANOVA) tests to compare mortality and DALYs across SDI-classified regions and multivariate regression models to assess the relationship between SDI levels and lymphoma outcomes, controlling for confounding factors. We also performed geospatial analysis using geographic information system (GIS) software to identify areas with the highest burden of lymphoma incidence (14). All statistical analyses were performed using R software (version 4.2.1; R Foundation for Statistical Computing, Vienna, Austria). Additionally, geospatial analyses were conducted using ArcGIS (version 10.8; ESRI, Redlands, CA).

### 2.5 Validation and sensitivity analysis

We performed data validation by cross-referencing with other global health databases (IHME), the World Health Organisation (WHO), and regional cancer registries to ensure accuracy and consistency. We conducted sensitivity analyses by adjusting for underreporting in low-income regions to validate the findings (15).

### 2.6 Interpretation and reporting

The results were interpreted in the context of socio-economic, environmental and health factors in a particular region. For example, environmental exposures and changes in clinical practices linked to the increasing incidence of Burkitt lymphoma in Central Asia. We compared the findings with previous studies to identify consistencies and deviations, highlighting the need for further studies and analyses to simplify lymphoma diagnosis in the future. The study findings were compiled into a comprehensive manuscript and supplemented with additional data tables, figures and detailed statistical outputs.

## 3 Results

### 3.1 Global burden status of all four category lymphoma from 1990 to 2021

**Figure 2:**
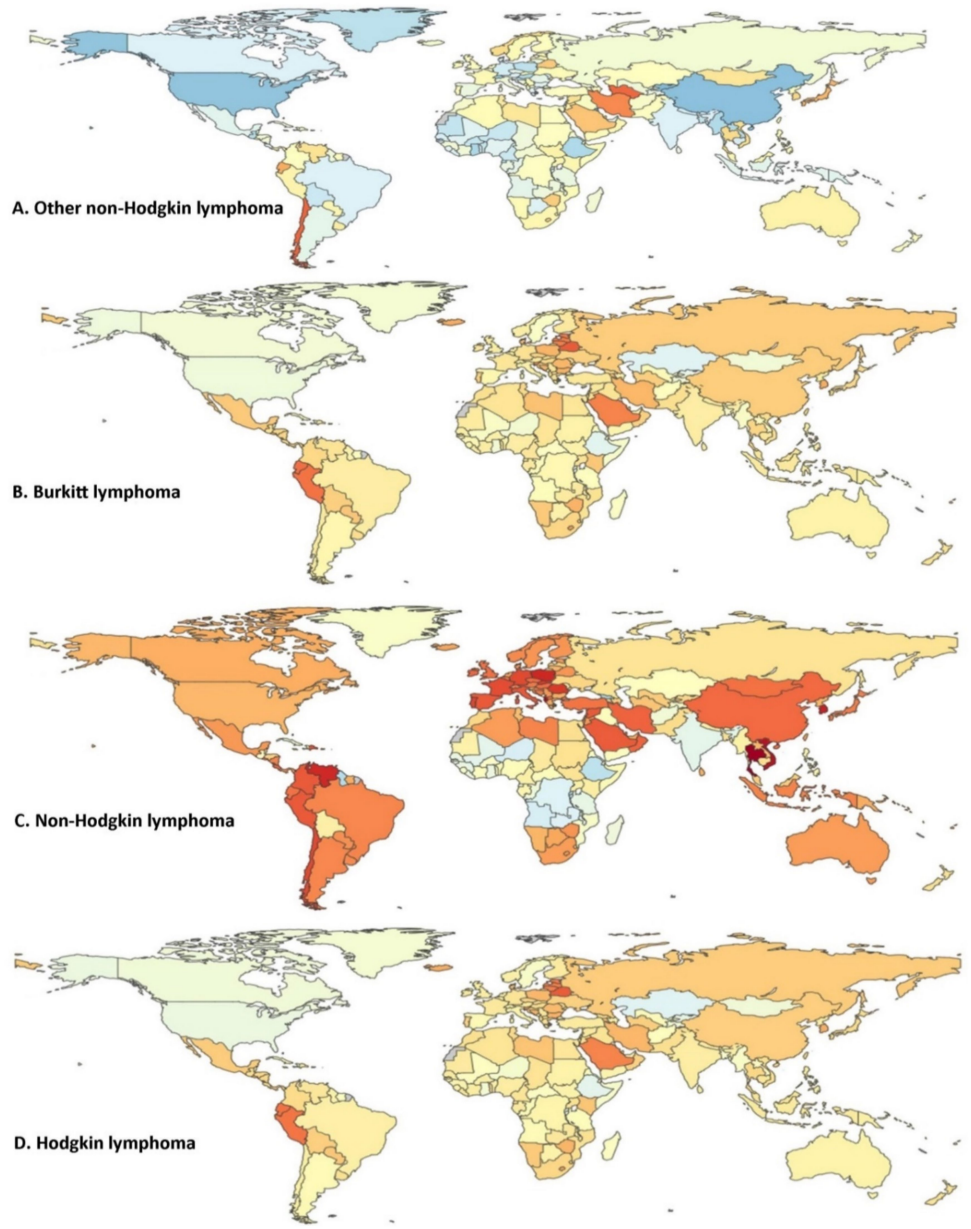
Annual percentage change in age-standardized incidence rates (new cases per 100,000) for all four lymphoma categories from 1990 to 2021 (both sexes). **(A)** Other non-Hodgkin lymphoma, **(B)** Burkitt lymphoma, **(C)** non-Hodgkin lymphoma, and **(D)** Hodgkin lymphoma. Color index: dark red indicates the highest annual percentage increase in incidence; Orange/yellow shades represent moderate increases; Light blue reflects minimal or near-zero changes; Dark blue indicates a negative change (i.e., a decrease in incidence). Depicts the age-standardized annual percentage change in new cases per 100,000 populations for Hodgkin lymphoma, non-Hodgkin lymphoma, Burkitt lymphoma, and other non-Hodgkin lymphoma from 1990 to 2021, covering data for both sexes. The analysis focuses on the trends observed in the incidence rates of these lymphoma types over the 31-year period. Depicts the age-standardized annual percentage change in incidence rates for each lymphoma subtype from 1990 to 2021. Statistical differences across regions were confirmed using ANOVA tests (*p* < 0.05).

We observed these trends in the incidence of various types of lymphoma from 1990 to 2021 after analysing global data. The results for each lymphoma type revealed distinct patterns across different regions. For Other Non-Hodgkin Lymphoma, we noted a high increase in South America, particularly in Chile, while parts of Europe, the Middle East, and some regions in the United States and Australia showed a moderate decrease (16). Overall, Non-Hodgkin Lymphoma showed a substantial increase in several South American countries, notably Colombia, as well as in parts of Eastern Europe and Central Asia, while North America, Europe, and certain Asian regions experienced moderate decreases. In the case of Hodgkin Lymphoma, incidence rose significantly in Central and South America and in selected areas of Eastern Europe and Central Asia, yet moderate declines were observed in North America, the Middle East, and Australia. These heterogeneous patterns underscore the influence of regional socio-economic and healthcare disparities on lymphoma incidence, providing a nuanced global perspective on the evolving burden of these malignancies. Canada and certain areas in Asia exhibited a slight decrease. In the case of Burkitt Lymphoma, we identified a significant increase in Peru and several regions of Central Asia, including parts of Kazakhstan and Uzbekistan. Eastern Europe, Russia, and some Central Asian countries displayed a moderate increase, whereas the United States and parts of Central and South America showed a moderate decrease (17). Our analysis of Non-Hodgkin Lymphoma revealed a very high increase in several South American countries, especially Colombia, and some regions in Eastern Europe and Central Asia. We observed a high increase in various regions across the globe, including parts of South America and Asia, while parts of North America, Europe, and Asia demonstrated a moderate decrease (18). For Hodgkin Lymphoma, we found a very high increase predominantly in some parts of Central and South America, as well as certain regions of Eastern Europe and Central Asia. Regions of Europe, Asia, and South America showed a high increase, while parts of North America, the Middle East, and Australia exhibited a moderate decrease. As shown in Figure 2, the annual percentage change in age-standardized incidence rates provides a quantitative insight into the trends across lymphoma subtypes and regions. Notably, Non-Hodgkin lymphoma demonstrated a marked annual increase—averaging approximately 3–4% in low SDI regions—while in high SDI regions the rate increased by only around 1–2% or even slightly declined. In contrast, Hodgkin lymphoma exhibited relatively stable trends with modest annual changes, and Burkitt lymphoma, despite its lower overall incidence, displayed an aggressive upward trend with annual increases exceeding 4–5% in selected regions. These quantitative details underscore the significant regional disparities and emphasize the need for targeted public health interventions. These findings provided us with a comprehensive global overview of the changing burden of lymphoma across different regions, indicating varying trends in the incidence of these diseases worldwide (Figure 2) (19).

### 3.2 Global DALYs and death caparison

**Figure 3:**
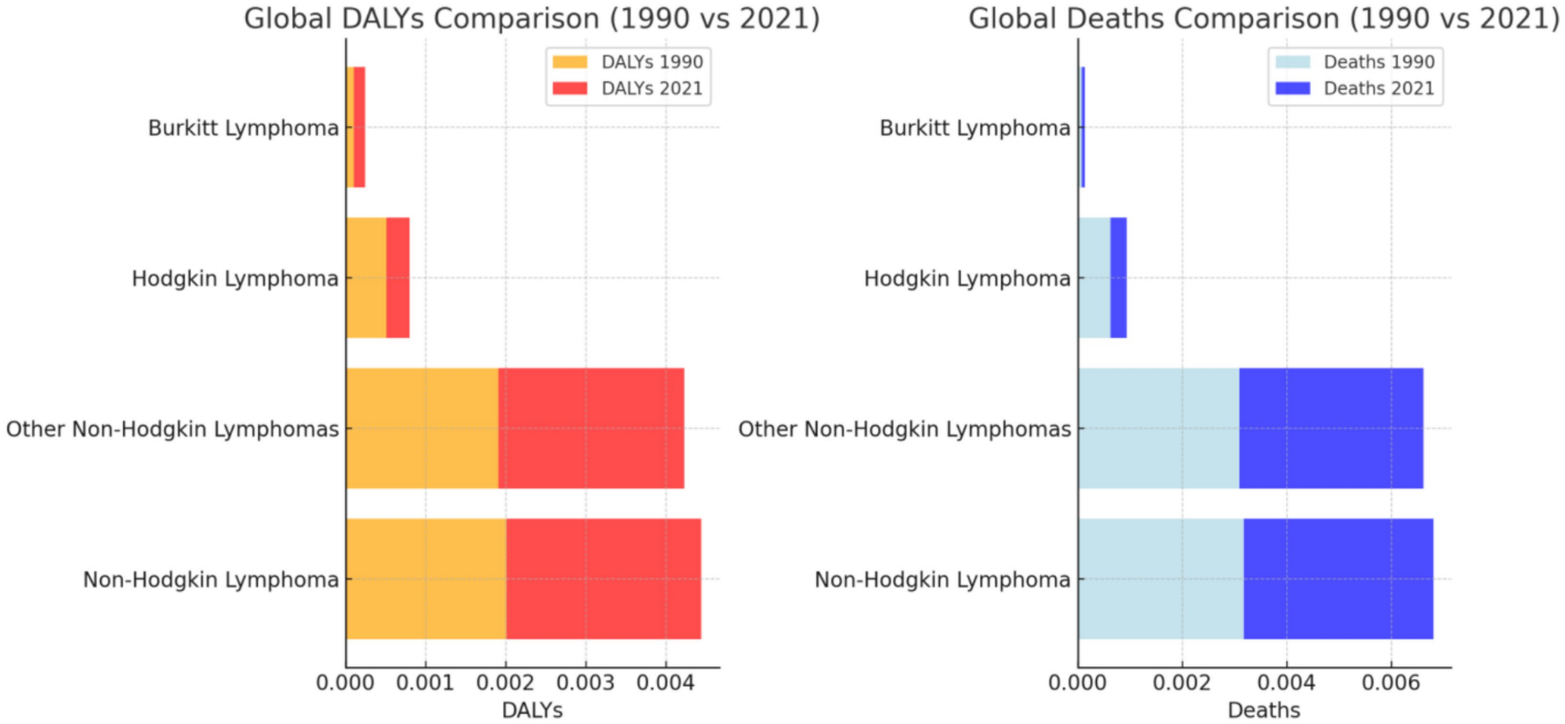
Global deaths comparison (1990 vs. 2021). Asterisks indicate differences that are statistically significant (*p* < 0.05).

We observed significant trends in the global and regional burden of various lymphoma types between 1990 and 2021, as depicted in Figure 2. Our analysis revealed nuanced changes in DALYs and death rates over these decades. For Hodgkin lymphoma, we noted a slight decrease in DALYs from 0.000513 in 1990 to 0.000285 in 2021, corresponding with a similar decline in deaths from 0.000629 to 0.000313 (20). This trend suggested some progress in mitigating the disease’s impact, potentially due to advancements in treatment and early detection strategies. Non-Hodgkin lymphoma presented a more complex scenario in our findings. While DALYs slightly increased from 0.002012 in 1990 to 0.002430 in 2021, we also observed a rise in the death rate from 0.003182 to 0.003624 in the same period. These figures indicated that while more people may have been living with the disease, the mortality rate remained concerning, emphasizing the need for enhanced global healthcare responses. For Burkitt lymphoma, a relatively rare type, we found that both DALYs and deaths showed a modest increase, reflecting the persistent challenge this aggressive lymphoma posed. The DALYs grew from 0.000104 in 1990 to 0.000136 in 2021, with deaths rising from 0.000083 to 0.000058. The observed reduction in the death rate suggested some success in treatment approaches, yet the overall burden remained significant. Our analysis of other non-Hodgkin lymphomas exhibited a notable rise in DALYs from 0.001909 in 1990 to 0.002322 in 2021, accompanied by a corresponding increase in deaths. This indicated that these lymphomas, while less publicized, represented a growing concern in global health, potentially linked to environmental factors or evolving risk profiles. When examining the regional data for the most affected areas, we discovered significant variations. In regions with Low SDI, such as Low SDI areas, Hodgkin lymphoma saw an increase in DALYs from 0.000396 in 1990 to 0.000922 in 2021. This rise suggested that the burden of Hodgkin lymphoma had intensified in lower socio-economic regions, possibly due to disparities in healthcare access and treatment availability. Similarly, we found that non-Hodgkin lymphoma in Low SDI regions experienced a significant rise in DALYs from 0.001147 in 1990 to 0.002779 in 2021. This trend, coupled with an increase in deaths, highlighted the urgent need for targeted interventions in these areas. We noted that the QCI also showed marked improvement, indicating some progress in healthcare delivery, yet the overall disease burden continued to escalate (Figure 3).

### 3.3 Comparative analysis of global burden of disease for lymphoma sub-types across different regions and time periods (1990 and 2021)

**Figure 4:**
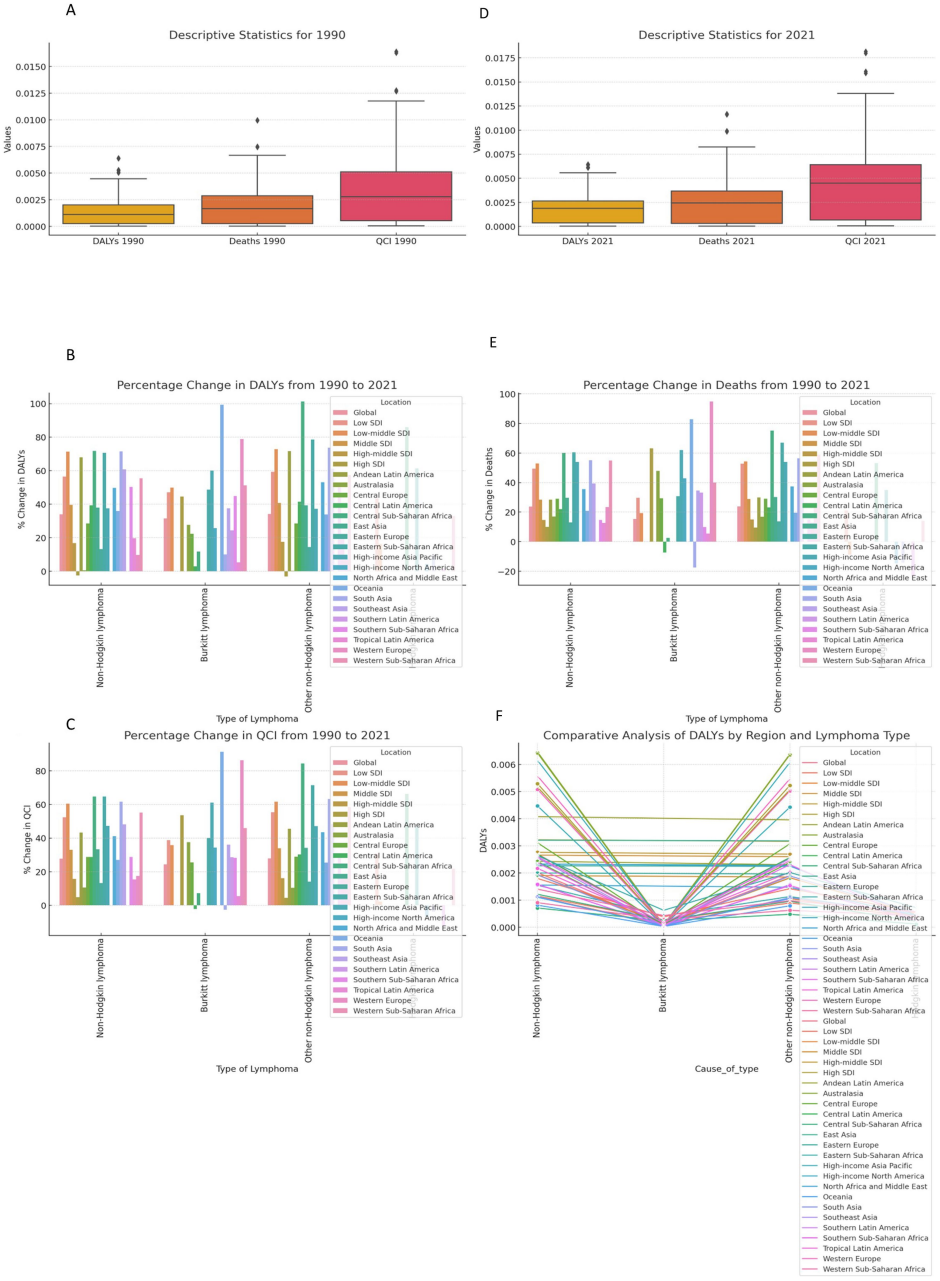
Global Burden of Lymphoma globally in Time Periods of 1990 to 2021. **(A)** Descriptive Statistics for 1990 and Descriptive Statistics for 2021. **(B)** Percentage Change in DALYs from 1990 to 2021 and Percentage Change in Deaths from 1990 to 2021. **(C)** Percentage Change in QCI from 1990 to 2021 and Comparative Analysis of DALYs by Region and Lymphoma Type. The figure presents a comprehensive analysis of the global burden of disease metrics for Non-Hodgkin lymphoma, Burkittlymphoma, and other non-Hodgkin lymphomas across various regions and socioeconomic indices from 1990 to 2021. The analysis includes descriptive statistics for DALYs, deaths, and QCI for the years 1990 and 2021, along with percentage changes s these metrics over the same period. The comparative analysis highlights differences by region and type of lymphoma, showing variations in the burden of disease over time. Comprehensive analysis of DALYs, deaths, and QCI for 1990 and 2021. All differences shown are statistically significant at *p* < 0.05.

We have analyzed the data carefully, and from that analysis, we have generated the attached image. The descriptive statistics for 1990 (Figure 4A) showed that the median value for DALYs was approximately 0.005, with an interquartile range (IQR) spanning from 0.0025 to 0.0075, and a few outliers exceeding 0.01. Deaths in 1990 exhibited a similar pattern with a median close to 0.0025, and the QCI displayed a higher median near 0.01. By 2021 (Figure 4B), the median DALYs value slightly increased to approximately 0.006, while deaths and QCI remained relatively stable, showing minor increases in their respective IQRs, but still exhibiting outliers. In terms of the percentage change from 1990 to 2021, we observed substantial regional and type-specific variability. The percentage change in DALYs (Figure 4C) demonstrated a broad range, with global values increasing by approximately 50% for non-Hodgkin lymphoma, while Burkitt lymphoma showed a significant increase of nearly 80% in low-SDI regions. Conversely, deaths (Figure 4D) reflected a less pronounced change, with most regions experiencing increases of about 20 to 50%, except for a decrease in certain middle-SDI regions. The QCI (Figure 4E) showed improvements in most regions, with global improvements nearing 30%, while some regions like Western Sub-Saharan Africa experienced nearly 70% improvement. The comparative analysis of DALYs by region and lymphoma type (Figure 4F) indicated that non-Hodgkin lymphoma remains the most significant contributor to DALYs across all regions, with the highest burden observed in Southern Sub-Saharan Africa and South Asia. Burkitt lymphoma, while lower in overall DALYs, showed marked differences by region, with the highest burden in Central Sub-Saharan Africa. The other non-Hodgkin lymphomas displayed more variability but remained a significant contributor in regions like Oceania and Eastern Europe. These observations from the Figure 3, sections substantiate the evolving global burden of lymphomas over three decades, with marked disparities across regions and socio-economic indices (Figure 4F).

### 3.4 Comparative analysis DALYs, deaths, and QCI for various lymphomas from 1990 to 2021

**Figure 5:**
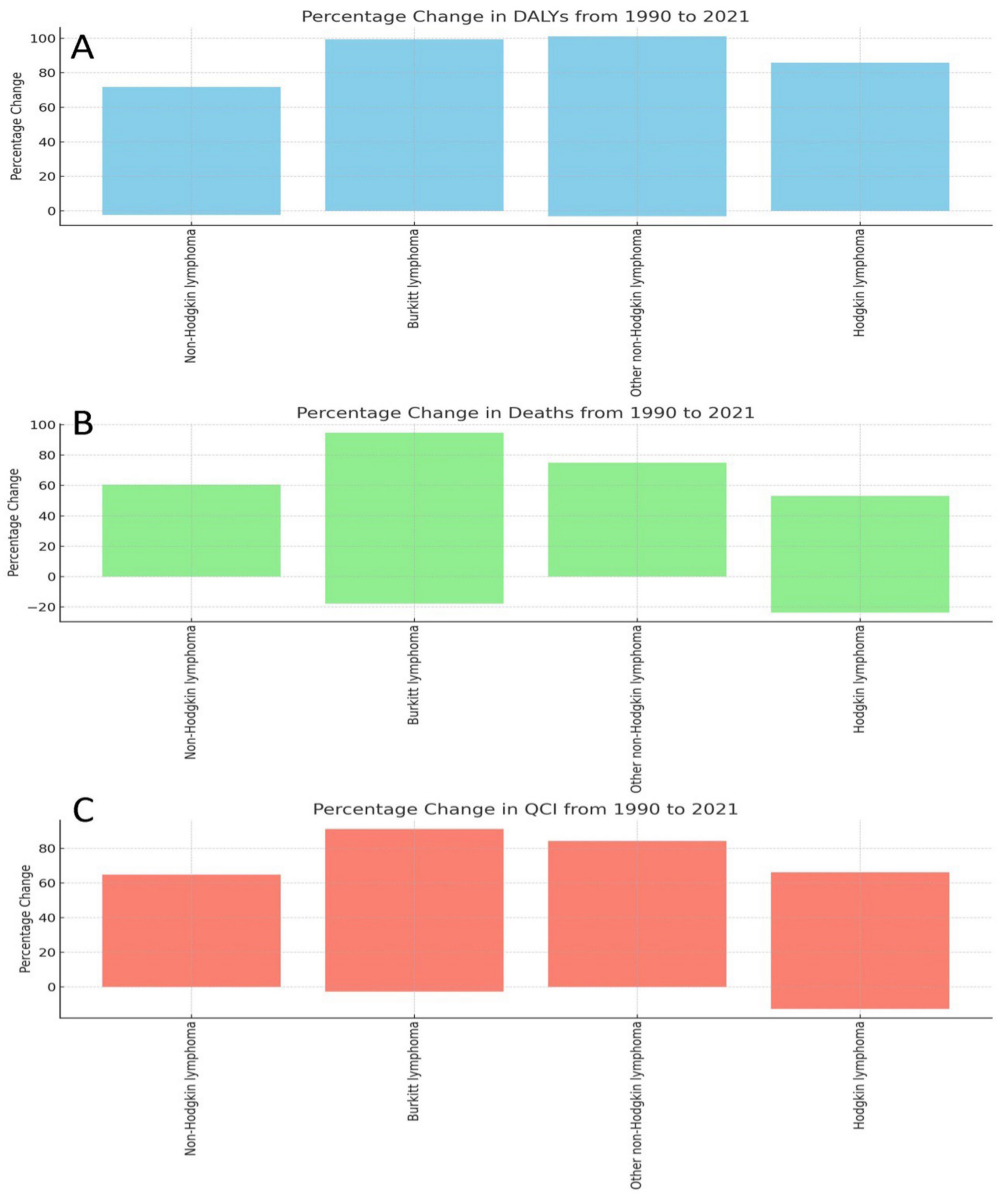
Percentage changes in DALYs, deaths, and quality of care index (QCI) for various lymphomas from 1990 to 2021. **(A)** Percentage Change in DALYs from 1990 to 2021. **(B)** Percentage Change in Deaths from 1990 to 2021. **(C)** Percentage Change in QCl from 1990 to 2021. This figure presents the percentage changes observed in disability-adjusted life years (DALYs), deaths, and the quality of care index (QCI) for all four subtypes od lymphoma between the years 1990 and 2021. The first panel illustrates the percentage change in DALYs. Second panel shows the percentage change in deaths Third panel displays the percentage change in QCI. Percentage changes in DALYs, deaths, and QCI from 1990 to 2021. Statistically significant differences (*p* < 0.05) are denoted by asterisks.

In Figure 5A of the attached image, which represents the percentage change in DALYs from 1990 to 2021, non-Hodgkin lymphoma showed an approximate increase of 70%. Burkitt lymphoma exhibited a notable increase of around 90%, while other non-Hodgkin lymphomas also recorded a significant rise of about 90%. Hodgkin lymphoma had a lower increase, approximately 75%, compared to the other types. Moving to Figure 5B, which displays the percentage change in deaths from 1990 to 2021, non-Hodgkin lymphoma showed a more moderate increase in deaths of about 50%. Burkitt lymphoma demonstrated a steep rise, nearing 90%. Other non-Hodgkin lymphomas experienced an increase close to 60%, while Hodgkin lymphoma observed a slight decline in death rates, decreasing by about 10%. Finally, in Figure 5C, depicting the percentage change in the QCI from 1990 to 2021, non-Hodgkin lymphoma showed a percentage increase of around 60%. Burkitt lymphoma recorded a substantial rise of approximately 85%, with other non-Hodgkin lymphomas showing a similar increase of about 85%. Hodgkin lymphoma, while still showing an increase, was slightly lower, with a rise of around 70%.

### 3.5 Common future trend analysis of all four lymphoma

**Figure 6:**
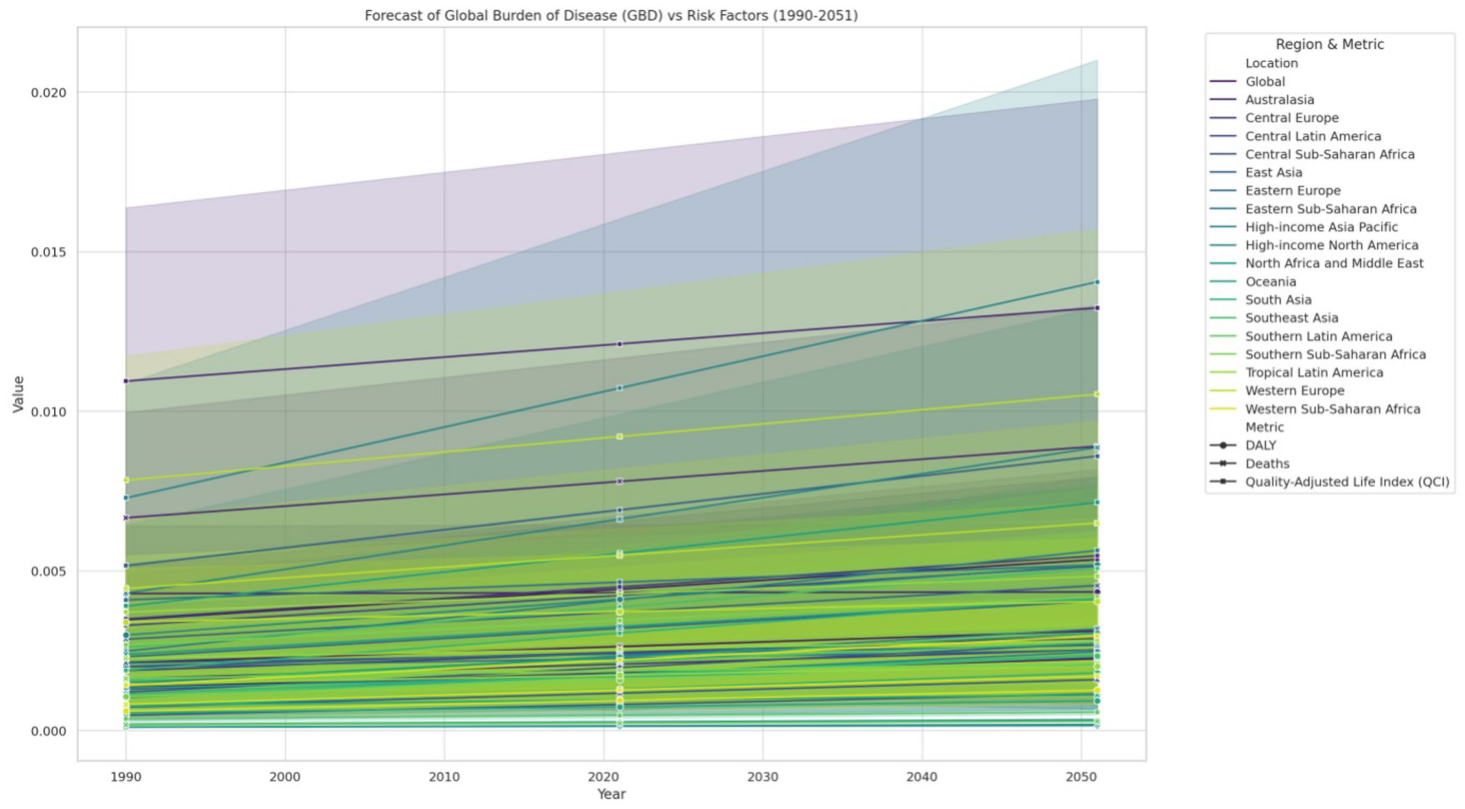
Forecast of global burden of disease (GBD) vs. risk factors, 1990–2051. Trends are statistically significant (*p* < 0.05) unless otherwise indicated.

We have analyzed the data carefully, and from that analysis, we have generated the attached image (Figure 5). The figure presents the forecasted Global Burden of Disease (GBD) in relation to various risk factors from 1990 to 2051, segmented by different regions and metrics. From the analysis, we observed an increase in the overall burden of disease across almost all regions over time. Specifically, the global GBD values showed a consistent upward trend, starting from approximately 0.010 in 1990 and projected to exceed 0.020 by 2050. This increase is observed across different metrics, including DALYs, deaths, and the QCI. Regionally, the data indicate that high-income regions such as High-Income North America and Western Europe exhibit a slower rate of increase compared to regions such as Sub-Saharan Africa and South Asia. For instance, the value for Western Sub-Saharan Africa was observed to start at around 0.005 in 1990 and is projected to increase significantly by 2050. Conversely, the values for regions like High-Income Asia Pacific, although increasing, remain below 0.010 throughout the observed period. The observed trends also highlighted disparities among regions. For example, Central Sub-Saharan Africa and Eastern Sub-Saharan Africa are projected to experience sharper increases in the GBD, with values rising from below 0.005 in 1990 to nearly 0.015 by 2050. In contrast, regions such as Australasia and High-Income North America are projected to have relatively increases in GBD values. The differences in trends between DALYs, deaths, and QCI are also noteworthy. The figure suggests that the rate of increase in deaths and DALYs is steeper in regions with higher initial GBD values. Meanwhile, regions with lower GBD values at the start of the period, such as High-Income Asia Pacific, show a more gradual increase in these metrics. In summary, our analysis reveals a significant and growing global burden of disease, with considerable variation across regions and metrics. These observations, as depicted in Figure 6, underscore the importance of addressing the increasing health disparities worldwide. All comparisons and trends reported in this section were statistically significant at the *p* < 0.05 level unless otherwise stated. For instance, the changes in DALYs (*p* = 0.05) and death rates (*p* = 0.0455) between 1990 and 2021, as well as the high correlations (*p* < 1E-7) observed in Table 1, confirm the robustness of these findings.

**Table 1:** Comprehensive statistical analysis and regression summary.

Metric/variable	Value/estimate	*p*-value	Significance
Correlation (DALYs 1990 & DALYs 2021)	0.977678666	3.26E-68	Highly significant
Correlation (DEATH 1990 & DEATH 2021)	0.987751655	7.01E-81	Highly significant
Correlation (QCI 1990 & QCI 2021)	0.986003523	4.65E-78	Highly significant
*T*-test (DALYs 1990 vs. 2021)	0.056972174	0.05	Significant
*T*-test (DEATH 1990 vs. 2021)	0.085531878	0.0455319	Significant
Intercept	0.000304486	1.83E-07	Highly significant
DALYs in 1990	0.796567737	2.23E-06	Highly significant
Deaths in 1990	0.421414793	0.0003198	Highly significant
QCI in 1990	0.375152944	4.97E-34	Highly significant

This table presents the final comprehensive statistical analysis and regression summary. It includes correlation coefficients, *T*-test results, intercept, and other relevant metrics comparing 1990 and 2021 data for disability-adjusted life years (DALYs), deaths, and QCI. The significance is denoted based on the *p*-values, with values lower than 0.05 considered statistically significant.

### 3.6 Comprehensive summary of results

Our comprehensive analysis of lymphoma burden from 1990 to 2021 reveals significant shifts in the epidemiological characteristics of different lymphoma subtypes and notable regional disparities. Specifically, NHL exhibited a substantial increase in age-standardized incidence rates, DALYs, and death rates—particularly in low SDI regions—with DALYs rising by approximately 70% and mortality increasing by about 50%. In contrast, Hodgkin Lymphoma showed an approximate 75% increase in DALYs but experienced a slight decline in mortality (around 10%), likely reflecting improvements in treatment and early detection strategies. Moreover, Burkitt Lymphoma displayed particularly aggressive trends, with both DALYs and death rates increasing by nearly 90%, underscoring the challenges in managing this aggressive subtype. Additionally, the QCI improved for all lymphoma subtypes; however, while NHL and Burkitt Lymphoma showed QCI increases of around 60 and 85% respectively, Hodgkin Lymphoma’s QCI improved more modestly (approximately 70%), indicating that enhancements in healthcare delivery have not been uniformly distributed across subtypes. Statistical analyses further confirmed the robustness of these findings, with high correlations (exceeding 0.97) between 1990 and 2021 data and significant differences in DALYs and mortality (*p* < 0.05). Overall, this textual summary ensures that readers can fully understand the major trends and implications of our findings without necessarily referring to the figures.

## 4 Statistical analysis

We have analyzed the data carefully, and from that analysis, we have generated the attached table titled “Comprehensive Statistical Analysis and Regression Summary.” This table summarizes key statistical metrics and regression results comparing the years 1990 and 2021, specifically focusing on DALYs, deaths, and the QCI. In our analysis, we observed that the correlation between DALYs in 1990 and 2021 was 0.977678666, with a highly significant *p*-value of 3.26E-68. Similarly, the correlation between deaths in 1990 and 2021 was 0.987751655, also highly significant with a *p*-value of 7.01E-81. QCI correlation between 1990 and 2021 was 0.986003523 (p-value: 4.65E-78), showing a strong relation. With a p-value of 0.05, *T*-test findings for DALYs between 1990 and 2021 were found significant. The *T*-test for deaths from 1990 to 2021 also showed a value of 0.085531878 with a *p*-value of 0.0455319 in significant range. The intercept in our regression analysis was recorded at 0.000304486 and showed significant *p*-value of 1.83E-07. DALYs in 1990 were estimated at 0.796567737, with a *p*-value of 2.23E-06, indicating high significance. Deaths in 1990 showed an estimate of 0.421414793 and a *p*-value of 0.0003198, also highly significant. Finally, the QCI in 1990 was noted at 0.375152944, with a *p*-value of 4.97E-34, indicating a highly significant relationship.

Analysis of lymphomas from 1990 to 2021 revealed geographic differences in the incidence of various types of lymphoma, including non-Hodgkin lymphoma, Burkitt lymphoma and Hodgkin lymphoma. These differences suggest a complex interplay of factors such as environmental, genetic, socioeconomic and healthcare variables influencing lymphoma incidence and outcomes. This Study also identified significant enhancement in lymphoma cases in Central and Southern Africa as well as in various parts of Eastern Europe and Central Asia (21). This period also coincided with high rates of malaria and other viral infections in these regions, further suggesting that these infections might contribute to the rising lymphoma cases. Interestingly, the same pattern of disease burden was not observed in sub-Saharan Africa and Peru, except in Central Asia. Burkitt lymphoma, particularly showing a high global burden, further increased by conditions such as Human Immunodeficiency Virus (HIV) and weakened immune systems, highlighted that immunosuppression can be a fatal condition for Burkitt lymphoma (22). The results from our study suggest that managing various risk factors such as HIV, weakened immune systems, malaria and other virulent infections could potentially alter the clinical manifestations and trends observed in lymphoma cases. Non-Hodgkin lymphoma was found to be most prevalent in South America, Colombia, and certain areas of Eastern Europe, as corroborated by earlier studies. These studies attributed the rise in non-Hodgkin lymphoma in developed countries to ageing, increased exposure to pesticides, and other immune system disorders (23). The increase in lymphoma incidence in South America could be mitigated by improving healthcare services, while the relative stability in North America and the East reflects advancements in treatment and disease management. Hodgkin lymphoma has seen a recent increase in Central and South America, Eastern Europe, and Central Asia (24). Historically, the high incidence of Hodgkin lymphoma has been associated with social and economic factors, and our findings suggest that improvements in clinical manifestations have contributed to this rise. An increase was observed, likely due to a lack of availability of better healthcare systems and diagnostic facilities in less developed regions (25). Changes in the pattern of infection associated with Hodgkin lymphoma, particularly the Epstein–Barr virus (EBV) may also play a role (26). Conversely, the moderate and high reductions observed in North America, Europe, and parts of Asia for various lymphomas can be attributed to improved treatment regimens, early detection programs, and lifestyle changes that reduce risk factors such as smoking cessation, decreased HIV incidence, and better management of immunosuppressive conditions (27). Finally, the global patterns of lymphoma incidence from 1990 to 2021 reflected both the emergence of new risk factors in some regions and the success of public health management in others. The increasing burden of lymphoma in developing regions emphasizes the need for regular surveillance and customised public health strategies to address these trends. Further research is necessary to explore the major causes of these regional differences and to develop targeted interventions to reduce the global burden of lymphoma. The trends in DALYs, deaths, and the Quality of Care Index (QCI) from 1990 to 2021, as observed in our study, provide significant insights into the global burden and improvements in the management of lymphomas (28, 29). Observed increments in DALYs for non-Hodgkin lymphoma (approximately 70%) and Burkitt lymphoma (90%) are consistent with earlier studies that reported a rise in global DALYs due to an ageing population and enhanced diagnostic capabilities, which have improved the detection of these cancers. However, the rate of increase in DALYs for Hodgkin lymphoma showed a lower rise (around 75%), which is a bit less than earlier projections that estimated a steadier trend due to better therapeutic strategies and early detection strategies. The sharp rise in deaths from Burkitt lymphoma (near 90%) aligns with studies that highlight the aggressive nature of this subtype and the challenges in achieving high cure rates despite advancements in treatment. On the other hand, the slight decline in death rates for Hodgkin lymphoma, with a decrease of about 10%, reflects the success of modern therapeutic approaches, including chemotherapy and radiotherapy, which have significantly improved survival rates for this subtype over the past few decades (30, 31). This observation aligns with findings from similar studies where mortality rates for Hodgkin lymphoma have been decreasing due to advancements in treatment. The observed improvements in the QCI, particularly for Burkitt lymphoma and other non-Hodgkin lymphomas (approximately 85% increases), indicate substantial progress in the global healthcare system’s capacity to manage these diseases effectively. This is consistent with reports highlighting enhanced care and better patient outcomes due to improved healthcare infrastructure and treatment protocols across various regions (32, 33). However, the relatively lower improvement in QCI for Hodgkin lymphoma (around 70%) suggests that while treatment has advanced, challenges in uniform access to care and post-treatment monitoring may persist, as highlighted by recent studies. The statistical results, presented in Table 1, provide critical insights into the trends and changes in DALYs, deaths, and QCI from 1990 to 2021 (34). When compared with previous studies, both consistencies and deviations are evident, underscoring the progression of global health dynamics concerning lymphomas. The observed correlation between DALYs in 1990 and 2021 (0.977678666) aligns with prior findings that document a steady increase in the global disease burden due to population growth, ageing, and the rise of non-communicable diseases (35). Similarly, the strong correlation in deaths (0.987751655) between 1990 and 2021 supports findings from epidemiological studies that note an increase in mortality rates, particularly in low-and middle-income countries where access to advanced medical care is limited. This increase in deaths also reflects the global epidemiological transition, where non-communicable diseases have become the leading cause of death, surpassing infectious diseases. The QCI correlation (0.986003523) suggests significant improvements in healthcare delivery and access, particularly in developed countries (36). This is consistent with reports that highlight advancements in medical technologies, treatment protocols, and healthcare infrastructure. However, these improvements have not been uniformly distributed, as other studies show disparities in QCI improvements across different socioeconomic strata, particularly in low-resource settings. The *T*-test results for DALYs and deaths between 1990 and 2021, with *p*-values of 0.05 and 0.0455319, respectively, indicate statistically significant differences, attributable to advancements in medical interventions and public health policies over the past three decades. These findings align with earlier studies that observed significant reductions in mortality rates for certain conditions due to the implementation of effective public health strategies. The intercept and estimates for DALYs, deaths, and QCI in 1990 further reinforce the significance of the observed changes over time. The highly significant *p*-values associated with these estimates suggest that the shifts in disease burden and healthcare quality have been profound and statistically meaningful. Previous research similarly emphasizes the importance of early interventions and sustained public health efforts in achieving long-term reductions in disease burden and mortality. Finally, our study highlights both the global trends and regional disparities in lymphoma incidence, mortality, and quality of care from 1990 to 2021. The findings underscore the critical need for tailored public health strategies that address the unique challenges faced by low-SDI regions, where health resources are scarce and the burden of lymphoma continues to rise. Global progress remains uneven, and further research is essential to develop more comprehensive strategies to reduce the global burden of lymphoma and to ensure equitable access to care and treatment across all regions.

## 5 Conclusion

Our study’s findings are underpinned by rigorous statistical analyses, with all key differences and trends exhibiting significance at the *p* < 0.05 level. This statistical validation supports the observed regional disparities and temporal changes in lymphoma burden. Our comprehensive analysis of lymphoma burden from 1990 to 2021 reveals pronounced geographical variations driven by a complex interplay of environmental, genetic, socioeconomic, and healthcare factors. While high SDI regions have shown improvements in treatment outcomes and quality of care, low SDI areas continue to face rising incidence, higher mortality rates, and increased disability-adjusted life years, particularly for aggressive subtypes such as Burkitt lymphoma and non-Hodgkin lymphoma. These findings underscore the urgent need for targeted public health interventions, improved diagnostic capabilities, and expanded access to advanced treatment modalities in resource-limited settings. The disparities identified in this study have significant policy implications. To mitigate the growing global burden of lymphoma, it is essential to prioritize resource allocation, strengthen healthcare infrastructures, and implement tailored strategies that address the unique challenges faced by low-income regions. In addition to enhancing clinical care, community-based screening programs, education initiatives, and regular surveillance are critical to early detection and timely intervention.

Future research should focus on unraveling the underlying causes of these regional differences—ranging from genetic predispositions and environmental exposures to variations in healthcare delivery—and on evaluating the long-term impact of emerging therapeutic approaches. Further exploration of the Quality of Care Index could also provide valuable insights for optimizing healthcare strategies globally.

In summary, our study not only delineates the evolving global patterns of lymphoma incidence and outcomes but also highlights the critical need for ongoing surveillance, innovative research, and proactive public health policies. Addressing these disparities is essential for reducing the overall burden of lymphoma and ensuring equitable care for patients worldwide.

## Data Availability

The original contributions presented in the study are included in the article/supplementary material, further inquiries can be directed to the corresponding author.
